# Relationship of postprandial fibroblast growth factor 21 with lipids, inflammation and metabolic dysfunction-associated fatty liver disease during oral fat tolerance test

**DOI:** 10.3389/fendo.2024.1343853

**Published:** 2024-05-17

**Authors:** Xiaolong Li, Kunjie Zheng, Lifang Liu, Tingxue Zhang, Wei Gu, Xiaoyu Hou, Jianlin Geng, Guangyao Song

**Affiliations:** ^1^ Department of Internal Medicine, Hebei Medical University, Shijiazhuang, Hebei, China; ^2^ Department of Endocrinology, Harrison International Peace Hospital, Hengshui, Hebei, China; ^3^ Department of Endocrinology, Hebei General Hospital, Shijiazhuang, Hebei, China; ^4^ Department of Endocrinology, Baoding First Central Hospital, Baoding, Hebei, China

**Keywords:** fibroblast growth factor 21, metabolic dysfunction-associated fatty liver disease, lipid, inflammation, oral fat tolerance test, postprandial state

## Abstract

**Introduction:**

Metabolic dysfunction-associated fatty liver disease (MAFLD) is closely associated with serum fibroblast growth factor (FGF) 21; however, previous studies have typically focused on the static fasting state, and the relationships between postprandial FGF21 levels, postprandial metabolic status, and MAFLD remain unclear. Therefore, we measured postprandial lipids, inflammatory factors, and FGF21 levels in MAFLD and further analyzed their relationship using an oral fat tolerance test (OFTT).

**Patients and methods:**

In total, 103 non-diabetic adult volunteers, including 46 patients with MAFLD, were included in this study. All participants underwent the OFTT. Venous blood samples were collected at 0, 2, 4, and 6 h. Circulating total cholesterol (TC), triglyceride (TG), free fatty acid (FFA), high-density lipoprotein cholesterol (HDL-C), low-density lipoprotein cholesterol (LDL-C), interleukin-6(IL-6), tumor necrosis factor-α(TNF-α), hypersensitive-C reactive protein(hs-CRP) and FGF21 were assessed.

**Results:**

Serum FGF21 significantly increased in the fasting state (*P* < 0.05) and showed a biphasic change of first decreasing and then increasing in MAFLD during the OFTT. The postprandial levels of TG, TC, LDL-C, FFA, IL-6, TNF-α and hs-CRP were significantly increased in MAFLD (*P* < 0.05). After adjusting for multiple factors, the FGF21 incremental area under the curve (iAUC) was linearly correlated with the FFA iAUC, TG iAUC, and IL-6 iAUC (*P* < 0.05) and was an independent factor for MAFLD (*P* < 0.05, OR=1.403).

**Conclusion:**

Dyslipidemia and excessive inflammation in MAFLD are associated to FGF21 levels in the postprandial period. An abnormal postprandial FGF21 response may be an important mechanism of MAFLD.

## 1 Introduction

Metabolic dysfunction-associated fatty liver disease (MAFLD), formerly named non-alcoholic fatty liver disease (NAFLD), is the most common chronic liver disease in Western countries and a major disease that seriously threatens the global public health (1). However, no specific drugs have been approved by the Food and Administration (FDA) for MAFLD. The pathophysiology of MAFLD is not yet fully understood, and hepatic metabolism is a dynamic process; however, current studies on MAFLD typically focus on the static fasting state.

Fibroblast growth factor (FGF) 21 is a powerful metabolic hormone primarily produced by the liver and acts in an endocrine manner. FGF21 is a core multifaceted regulator of glycolipid metabolism and inflammatory response (2). Previous studies have found that fasting FGF21 is strongly associated with liver fat content in a dose-dependent manner and is a biomarker of liver fat content in MAFLD (3). Non-alcoholic steatohepatitis is associated with fasting FGF21 levels (4). However, humans spend most of their day in the postprandial state, which is acknowledged as a complex interplay among nutrients, hormones, and diet-derived metabolites, and is characterized by the coexistence of metabolism and inflammation (5). The liver is one of the most important organs for regulating postprandial metabolism and hormonal responses in the human body. A recent study has shown that postprandial metabolic disorders exist in MAFLD (6). Particularly, postprandial response in healthy adults has been shown to be closely related to postprandial FGF21 changes (7). Nutrient intake plays a significant role in the regulation of FGF21 (8), and a high-fat diet is a widely prevalent dietary profile today, which is a major driver of metabolism-related disorders such as MAFLD. It is not clear how postprandial responses to a high-fat diet are associated with MAFLD and whether these are associated with changes in FGF21.

This study aimed to observe changes in postprandial status after oral high-fat meals in the MAFLD population and to further analyze the correlation between postprandial FGF21 and lipids, inflammatory factors, and MAFLD.

## 2 Materials and methods

### 2.1 Study sample

Volunteers were randomly recruited from the outpatient clinic of the Department of Endocrinology at Hebei Provincial People’s Hospital from November 2018 to December 2019, with participants aged between 18–65 years and with a of body mass index (BMI) ≥18 kg/m^2^. The Ethics Committee of Hebei General Hospital approved the research protocol, which conformed to the provisions of the Declaration of Helsinki. The study has been registered with the China Clinical Trial Registry (registration number: ChiCTR1800019514).

### 2.2 Exclusion criteria

The criteria for exclusion were as follows: digestive system diseases (diseases in the liver, gallbladder, pancreas, or spleen), infectious diseases, vegetarian diet, thyroid dysfunction, heart disease, blood diseases, malignant tumors, kidney diseases, psychosis, pregnancy, smokers, stroke, history of surgery and/or trauma, use of drugs (contraceptives, antibiotic, fish oil, hormone β receptor blockers, diuretics), and weight change >3 kg (3 months). Oral glucose tolerance test (OGTT) was performed in all eligible participants except diabetes mellitus. To avoid acute pancreatitis induced by the high-fat test and test errors caused by extreme values, subjects with fasting TG >5 mmol/L were excluded. The remaining participants underwent further oral fat tolerance test (OFTT).

### 2.3 Oral fat tolerance testing

The OFTT was conducted uniformly in all participants, and all participants were required to receive a standardized meal for 1 week before the OFTT and were prohibited from smoking and drinking alcohol. The formulation of the high-fat meal for the OFTT was made by the group with reference to the dietary guidelines for the Chinese population and was completed with the assistance of a professional dietitian. Each high-fat meal contained 1500 kcal, 60% fat (saturated fatty acids 20%, monounsaturated fatty acids: 40%, polyunsaturated fatty acids: 40%), 20% proteins, 20% carbohydrates. Food and water were fasted after 22:00 h on the night before the trial, followed by a standardized high-fat meal at 8:00 h the following day, with a 10-min meal and a 6-h fast, with free access to water but no smoking or strenuous activity. Venous blood was collected before and at 2, 4, and 6 h after the meal. Blood was immediately chilled and centrifuged. Subsequently, aliquots were immediately frozen at −80°C (Haier MDR-382E; Haier, Qingdao, China) until assayed.

### 2.4 Measurement of biochemical indicators

Fasting blood glucose (FBG), hs-CRP, TC, TG, FFA, HDL-C, and LDL-C levels before and after a high-fat meal were assayed using a fully automated biochemical analyzer manufactured by Hitachi, Japan. The serum insulin (FINS) were measured using electrochemiluminescence. The serum TNF-α was measured by ELISA kit(MUTISCIENCES, Hangzhou, China). Serum FGF21 and IL-6 were assayed using an enzyme-linked immunosorbent assay kit (R&D Systems, USA).

A homeostasis model was used to evaluate insulin resistance [HOMA-IR= FBG (mmol/L) × FINS (mIU/L)/22.5) (9). The body mass index (BMI) was calculated as the ratio of weight to height squared (kg/m^2^). Postprandial changes in serum FGF21, lipids, and inflammatory factors were measured as incremental area under the curves (iAUCs) during the OFTT compared to the fasting levels, which were calculated using the trapezoidal area method.

### 2.5 Diagnosis of MAFLD

MAFLD was diagnosed based on a new guidance from over 70 societies, including the American Association for the Study of Liver Diseases (AASLD), the American Gastroenterological Society (AGA), and the European Association for the Study of the Liver (EASL) (10). Hepatic steatosis was diagnosed based on liver ultrasound findings.

### 2.6 Statistical analysis

SPSS version (version 25.0, IBM Corp., Armonk, NY, USA) and GraphPad Prism software (version 8.0, San Diego, CA, USA) were used for the statistical analysis. Normally distributed measures were expressed using mean ± standard deviation (SD), and non-normal measures were expressed using the median [M(P25, P75)]. Normally distributed data between the two groups were compared by applying an independent samples t-test. Non-parametric tests should be applied to data that are not normally distributed or whose variances are not homogeneous. Two-factor repeated-measures analysis of variance (rmANOVA) was applied to assess postprandial changes in parametric indicators over time, as well as between-group differences with Greenhouse-Geisser correction. Pearson’s correlations were performed to detected the associations between normally distributed variables; otherwise, Spearman’s correlation coefficient was applied. Multiple linear regression analysis was used to assess the relationship between FGF21 iAUC and postprandial changes in lipid and inflammatory indicators. Binary logistic regression analysis was used to assess the association between the FGF21 iAUC and MAFLD. Statistical significance was set at *P* < 0.05 (two-tailed).

## 3 Results

### 3.1 Comparison of baseline information between the two populations

We recruited 103 participants: 57 in the control group and 46 in the MAFLD group. There were no significant differences in sex or age between the two groups (*P*>0.05). The median FGF21 level in the overall population was 266.38 pg/mL. In the MAFLD group, fasting serum FGF21 levels were significantly higher. In the MAFLD group, BMI, WC, SBP, DBP, TC, TG, FFA, LDL-C, FBG, FINS, HOMA-IR, IL-6, TNF-α, and hs-CRP were significantly higher, and HDL-C was significantly lower, which were all statistically different from Con group (*P* < 0.05) (**Table 1**). These data demonstrated the characteristics of the MAFLD population, including metabolic disorders, excessive inflammation, and higher FGF21 levels.

**Table 1:** Comparison of basic characteristics of participants in the two groups.

	Total (n=103)	Con (n=57)	MAFLD (n=46)	*P*
Age (years)	49.62 ± 11.49	47.68 ± 12.10	52.02 ± 10.31	0.056
Male, n (%)	51 (49.5%)	27 (47.4%)	24 (52.2%)	0.694
BMI (kg/m^2^)	26.84 ± 4.75	24.46 ± 3.81	29.78 ± 4.13	<0.001
WC (cm)	90.06 ± 12.81	84.00 ± 11.07	97.57 ± 10.74	<0.001
SBP (mmHg)	126.66 ± 17.23	120.56 ± 16.75	134.22 ± 14.78	<0.001
DBP (mmHg)	79.02 ± 10.35	75.11 ± 9.66	83.87 ± 9.11	<0.001
TC (mmol/L)	4.83 ± 1.03	4.60 ± 0.98	5.13 ± 1.02	0.009
TG (mmol/L)	1.68 ± 1.04	1.17 ± 0.68	2.31 ± 1.07	<0.001
FFA (mmol/L)	0.86 ± 0.54	0.67 ± 0.45	1.10 ± 0.55	<0.001
HDL-C (mmol/L)	1.29 ± 0.29	1.34 ± 0.28	1.22 ± 0.29	0.039
LDL-C (mmol/L)	3.08 ± 0.76	2.88 ± 0.72	3.32 ± 0.76	0.004
FBG (mmol/L)	5.28 ± 0.49	5.09 ± 0.43	5.50 ± 0.47	<0.001
FINS (uIU/mL)	10.41 (7.76,14.31)	8.03 (6.01,12.38)	13.62 (10.16,16.00)	<0.001
HOMA-IR	2.52 (1.72,3.39)	1.83 (1.32,2.90)	3.15 (2.60,4.00)	<0.001
FGF21 (pg/ml)	266.38 (188.04,466.32)	234.78 (170.69,410.80)	288.67 (204.34,550.11)	0.026
IL-6 (pg/ml)	1.77 ± 1.41	1.38 ± 0.87	2.26 ± 1.77	0.001
TNF-α (ng/dl)	2.15 ± 1.65	1.80 ± 1.51	2.58 ± 1.72	0.015
hs-CRP (mg/L)	2.33 ± 1.50	1.62 ± 0.82	3.21 ± 1.68	<0.001

Means ± SD for normally distributed variables or medians (interquartile range) for non–normally distributed variables. BMI, body mass index; DBP, diastolic blood pressure; FBG, fasting blood glucose; FFA, free fatty acid; FINS, fasting insulin; FGF21, fibroblast growth factor 21; HDL-C, high-density lipoprotein-cholesterol; HOMA-IR, homeostasis model assessment of insulin resistance; LDL-C, low-density lipoprotein-cholesterol; SBP, systolic blood pressure; TC, total cholesterol; TG, triglyceride; WC, waist circumference; IL-6, interleukin-6; TNF-α, tumor necrosis factor; hs-CRP, C-reactive protein.

### 3.2 Changes in FGF21 during OFTT

**Figure 1:**
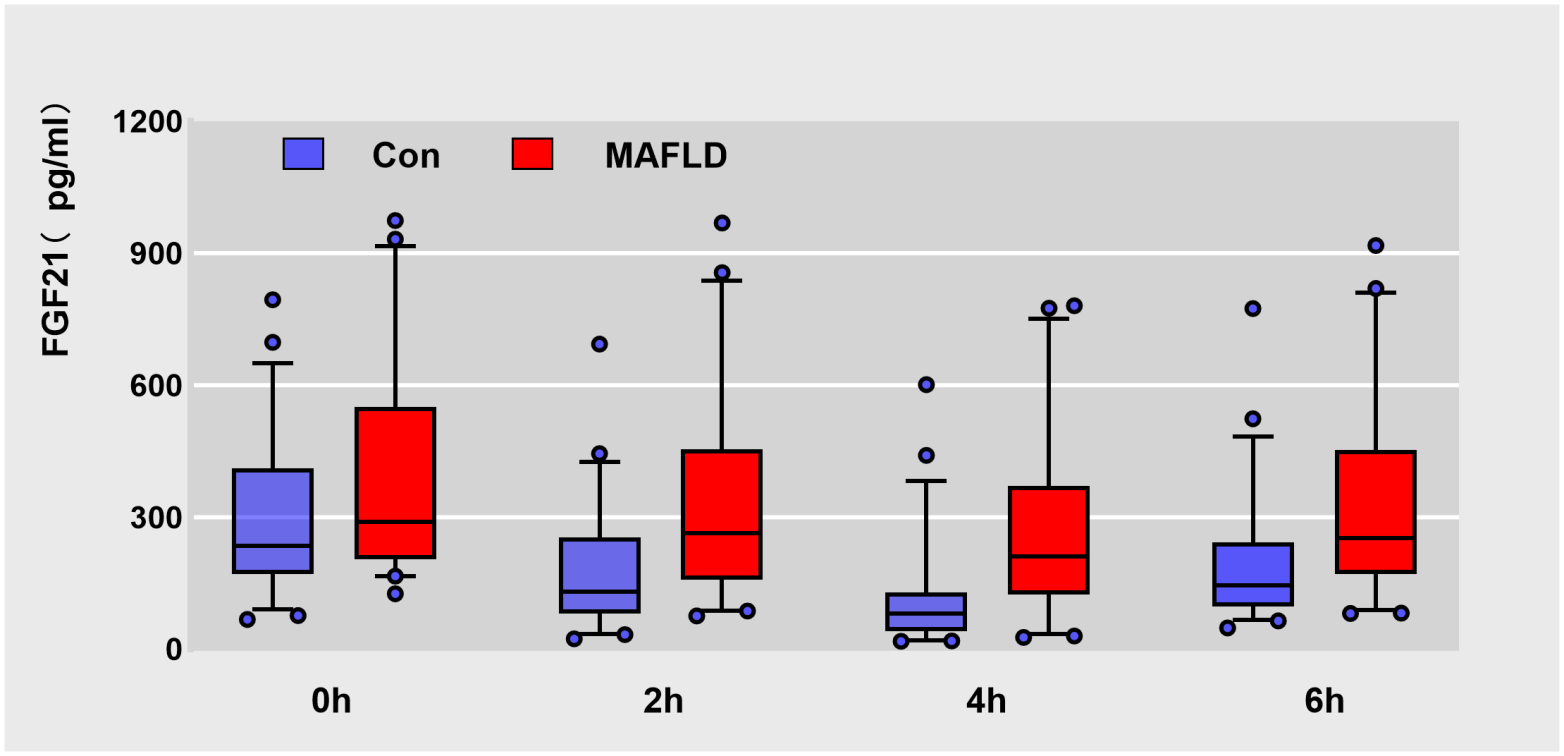
Box plots of raw data for Serum FGF21 levels at different time points during oral fat tolerance test. Data are median (central line), 5%-95% range (box margins), adjacent values (whiskers), and outliers (dots).

**Figure 2:**
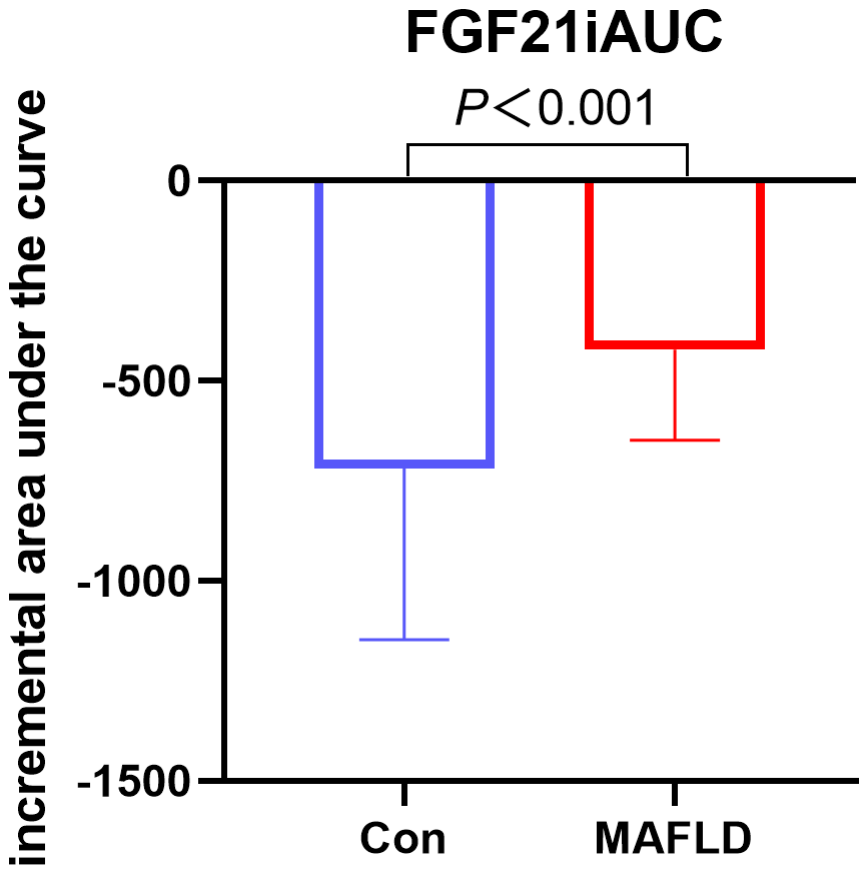
Changes in FGF21 after a high-fat meal in the two groups.

FGF21 showed a non-normal distribution, with a wide range of fluctuations. At all-time points, serum FGF21 levels were significantly higher in the MAFLD group (*P* < 0.05). The overall trend of FGF21 expression in both groups showed a biphasic change, first decreasing then increasing (**Figure 1**). Further analysis revealed that the FGF21 iAUC was significantly higher in the MAFLD group (*P* < 0.001) (**Figure 2**).

### 3.3 Changes in lipid levels during OFTT

**Figure 3:**
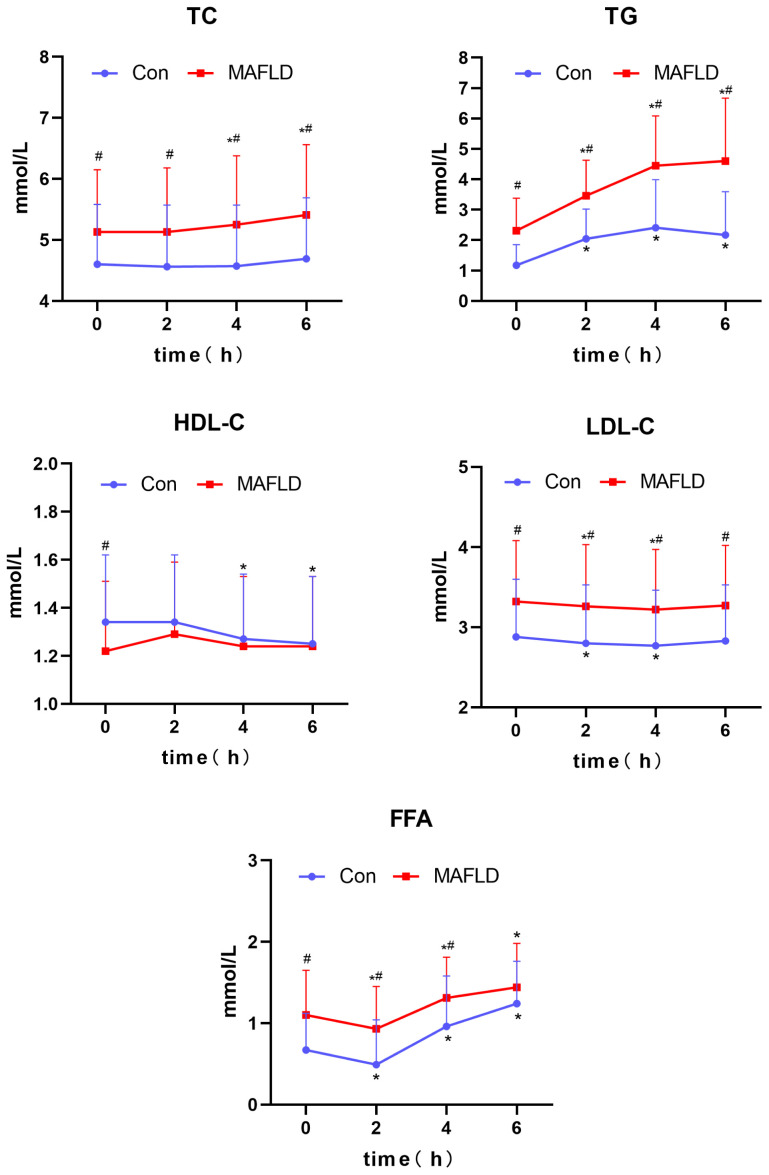
Changes in lipid levels after a high-fat meal in the two groups. TC, total cholesterol; TG, triglyceride; HDL-C, high-density lipoprotein-cholesterol; LDL-C, low-density lipoprotein-cholesterol; FFA, free fatty acid; iAUC, incremental area under the curve. *P < 0.05 versus 0 h in the same group, ^#^P < 0.05 versus Con group.

When the two populations were compared, there was no statistically significant difference in postprandial HDL-C (*P* > 0.05); except for FFA at 6 h, which was not statistically different (*P* > 0.05), serum TC, TG, LDL-C, and FFA levels at any time point were significantly higher in the MAFLD group than Con group (*P* < 0.05) (**Table 2**). During the OFTT, both groups showed the most significant changes in serum TG and FFA levels compared to baseline at 0 h. Postprandial TG levels increased significantly at 2 h in both groups (*P* < 0.05), with the Con group peaking at 4 h, and the MAFLD group not showing a peak. The FFA levels showed a biphasic change of decreasing and then increasing, and the trough time was 2 h, which was statistically different from that at 0 h (*P* < 0.05). TC, HDL-C, and LDL-C levels did not change significantly. TC and HDL-C levels in the MAFLD group showed significant upward and downward trends at 4 h, respectively (*P* < 0.05). LDL-C decreased in both groups at 2 and 4 h and was significantly different from that at 0 h (*P* < 0.05) (**Table 2**, **Figure 3**).

**Table 2:** Changes of lipids and serum inflammatory factors during OFTT in two groups.

	0 h	2 h	4 h	6 h
TC (mmol/L)				
Con	4.60 ± 0.98	4.56 ± 1.01	4.57 ± 1.00	4.69 ± 1.00
MAFLD	5.13 ± 1.02^#^	5.13 ± 1.05^#^	5.25 ± 1.13^*#^	5.41 ± 1.15^*#^
TG (mmol/L)				
Con	1.17 ± 0.68	2.04 ± 0.98^*^	2.41 ± 1.58^*^	2.17 ± 1.42^*^
MAFLD	2.31 ± 1.07^#^	3.46 ± 1.17^*#^	4.45 ± 1.63^*#^	4.60 ± 2.07^*#^
FFA (mmol/L)				
Con	0.67 ± 0.45	0.48 ± 0.48^*^	0.96 ± 0.62^*^	1.24 ± 0.52^*^
MAFLD	1.10 ± 0.55^#^	0.93 ± 0.52^*#^	1.31 ± 0.50^*#^	1.44 ± 0.54^*^
HDL-C (mmol/L)				
Con	1.34 ± 0.28	1.34 ± 0.28	1.27 ± 0.27^*^	1.25 ± 0.28^*^
MAFLD	1.22 ± 0.29^#^	1.29 ± 0.30	1.24 ± 0.29	1.24 ± 0.29
LDL-C (mmol/L)				
Con	2.88 ± 0.72	2.80 ± 0.73^*^	2.77 ± 0.69^*^	2.83 ± 0.70
MAFLD	3.32 ± 0.76^#^	3.26 ± 0.77^*#^	3.22 ± 0.75^*#^	3.27 ± 0.75^#^
IL-6 (pg/ml)				
Con	1.38 ± 0.87	1.88 ± 1.01^*^	2.49 ± 1.07^*^	2.42 ± 1.09^*^
MAFLD	2.26 ± 1.77^#^	2.82 ± 1.82^*#^	3.42 ± 1.75^*#^	4.14 ± 1.79^*#^
TNF-α (ng/dl)				
Con	1.80 ± 1.51	1.82 ± 1.63	1.81 ± 1.64	1.88 ± 1.66
MAFLD	2.58 ± 1.72^#^	2.43 ± 1.76^*^	2.44 ± 1.67	2.54 ± 1.70
hs-CRP (mg/l)				
Con	1.62 ± 0.82	1.66 ± 0.95	1.70 ± 0.97	1.69 ± 0.88
MAFLD	3.21 ± 1.68^#^	3.26 ± 1.76^#^	3.18 ± 1.81^#^	3.23 ± 1.68^#^

TC, total cholesterol; TG, triglyceride; HDL-C, high-density lipoprotein-cholesterol; LDL-C, low-density lipoprotein-cholesterol;FFA, free fatty acid; IL-6, interleukin-6; TNF-α, tumor necrosis factor; hs-CRP, C-reactive protein. *P < 0.05 versus 0 h in the same group, ^#^P < 0.05 versus Con group.

Further analysis revealed a significantly higher TG iAUC and lower FFA iAUC in the MAFLD group than in the control group (*P* < 0.001 and *P* < 0.05, respectively).

### 3.4 Changes in serum inflammatory factor levels during OFTT

**Figure 4:**
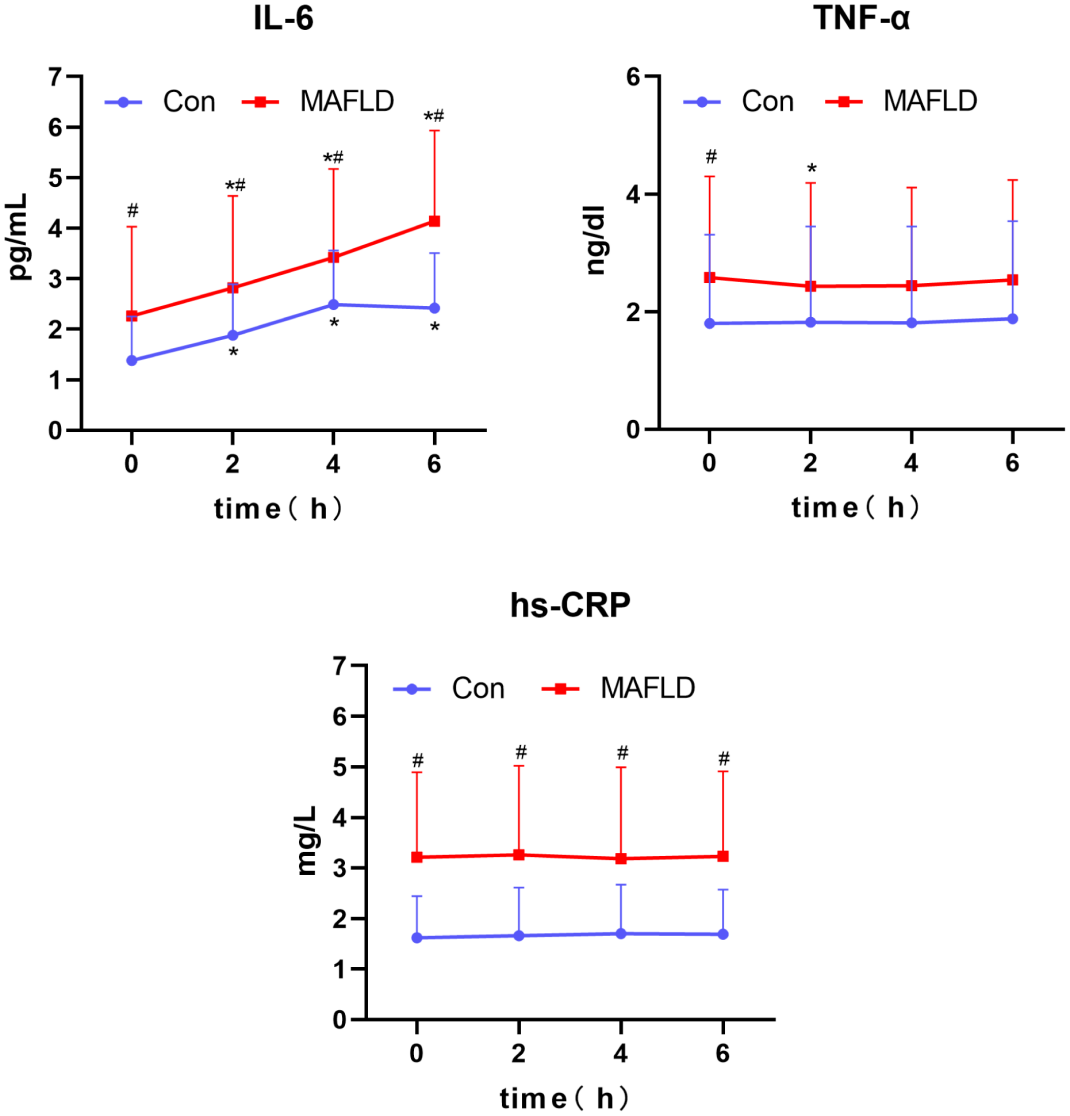
Changes in serum inflammatory factors after a high-fat meal in the two groups. IL-6, interleukin-6; TNF-α, tumor necrosis factor; hs-CRP, C-reactive protein. ^*^P < 0.05 versus 0 h in the same group, ^#^P < 0.05 versus Con group.

When comparing the two groups after a high-fat test meal, IL-6 and hs-CRP levels were significantly higher in the MAFLD group at any time point (*P* < 0.05), and TNF-α was significantly different only at fasting levels (*P* < 0.05). Compared with baseline levels at 0 h, IL-6 levels showed a significant increase at 2 h in both groups (*P* < 0.05), with the peak time occurring at 4 h in the control group and no peak in the MAFLD group. IL-6 iAUC was significantly higher in the MAFLD group than in the control group (*P* < 0.01). Compared with 0 h, changes in TNF-α and hs-CRP were not significant in both groups, except for TNF-α in the MAFLD group, which was statistically different at 2 h (*P* < 0.05) (**Table 2**, **Figure 4**).

### 3.5 Correlation of FGF21 iAUC with anthropometric and postprandial serologic indicators

Based on all subjects, the relationship between FGF21 iAUC and indexes were analyzed. The results showed that FGF21 iAUC was positively correlated with DBP, BMI, WC, TC iAUC, TG iAUC, IL-6 iAUC, and HOMA-IR (*P* all < 0.05) and negatively correlated with FFA iAUC (*P* < 0.05) (**Table 3**).

**Table 3:** Relationship between FGF21 iAUC and other indexes.

	FGF21 iAUC	
	r	*P*
Age	<0.001	0.996
SBP	0.188	0.058
DBP	0.212*	0.032
BMI	0.243*	0.013
WC	0.272*	0.005
TC iAUC	0.208*	0.035
TG iAUC	0.445*	<0.001
FFA iAUC	-0.274*	0.005
HDL iAUC	-0.014	0.891
LDL iAUC	0.02	0.843
IL-6 iAUC	0.45*	<0.001
TNF-α	-0.006	0.954
hs-CRP	0.192	0.052
HOMA-IR^a^	0.317*	0.001

BMI, body mass index; DBP, diastolic blood pressure; FFA, free fatty acid; FGF21, fibroblast growth factor 21; HDL-C, high-density lipoprotein-cholesterol; HOMA-IR, homeostasis model assessment of insulin resistance; LDL-C, low-density lipoprotein-cholesterol; SBP, systolic blood pressure; TC, total cholesterol; TG, triglyceride; WC, waist circumference; IL-6, interleukin-6; TNF-α, tumor necrosis factor; hs-CRP, C-reactive protein. r, correlation coefficient. a: Log transformed (base 10). *P<0.05.

### 3.6 Multiple linear regression analysis was used to clarify the relationship between FGF21 iAUC and postprandial lipid and inflammatory factor changes

Based on the results of the above correlation analysis, the relationship between changes in lipid and inflammatory factor levels and the FGF21 iAUC during the OFTT was further analyzed based on all subjects. Changes in the values of lipids (TC iAUC, TG iAUC, and FFA iAUC) and inflammatory factors (IL-6 iAUC) were respectively included in the regression model as independent variables and FGF21iAUC as dependent variable for multiple linear regression analysis, which showed that TG iAUC, FFA iAUC, and IL-6 iAUC were the influencing factors of FGF21 iAUC (*P* < 0.001, *P* < 0.05, *P* < 0.001). After adjusting for sex, age, SBP, and WC, TG iAUC, FFA iAUC, and IL-6 iAUC remained the influencing factors for FGF21 iAUC (*P* < 0.001, *P* < 0.05, *P* < 0.001, respectively). After further adjustment for HDL-C, LDL-C, HOMA-IR, IL-6, TG iAUC and FFA iAUC were found to independently influence FGF21 iAUC (*P* < 0.01, *P* < 0.05, respectively). After further adjustment for TG, HDL-C, LDL-C, and HOMA-IR, the IL-6 iAUC was found to have an independent influence on the FGF21 iAUC (*P* < 0.001) (**Table 4**).

**Table 4:** Multiple linear regression analysis of FGF21 iAUC.

Variable	Model 1		Model 2		Model 3	
	B (95% CL)	*P* value	B (95% CL)	*P* value	B (95% CL)	*P* value
TG iAUC	0.406 (0.219, 0.593)	<0.001	0.366 (0.170, 0.562)	<0.001	0.322 (0.121, 0.523)	0.002
FFA iAUC	-1.286 (-2.296, -0.276)	0.013	-1.318 (-2.341, -0.296)	0.012	-1.241 (-2.267, -0.215)	0.018
IL-6 iAUC	0.890 (0.542, 1.239)	<0.001	0.846 (0.500, 1.192)	<0.001	0.772 (0.412, 1.131)	<0.001

TC, total cholesterol; TG, triglyceride; FFA, free fatty acid; IL-6, interleukin-6; iAUC,incremental area under curve. Model 1: without adjustment. Model 2: Based on Model 1, further adjusted for sex, age, SBP and WC. Model 3 (TCiAUC, TG iAUC, and FFA iAUC as independent variables): Based on Model 2, further adjusted for HDL-C, LDL-C, HOMA-IR, IL-6. Model 3 (IL-6 iAUC as independent variables): Based on Model 2, further adjusted for TG, HDL-C, LDL-C, HOMA-IR.

### 3.7 Binary logistic regression analysis to clarify the relationship between FGF21 iAUC and MAFLD

Based on all subjects, binary logistic regression analysis performed with FGF21 iAUC as the independent variable and MAFLD as the dependent variable showed that FGF21 iAUC was an influencing factor of MAFLD (*P* < 0.001, OR=1.308). After adjusting for sex, age, SBP, and WC, the FGF21 iAUC remained an influencing factor for MAFLD (*P* < 0.01, OR=1.306). After further adjustment for TG, HDL-C, LDL-C, HOMA-IR, and IL-6, FGF21 iAUC was found to be an independent influencing factor for MAFLD (*P* < 0.05, OR=1.403) (**Table 5**).

**Table 5:** Binary logistic regression analysis to assess the risk of MAFLD.

Characteristic	OR	95% CL	*P value*
Modle 1			
FGF21 iAUC	1.308	1.129, 1.517	<0.001
Modle 2			
FGF21 iAUC	1.306	1.088, 1.568	0.004
Modle 3			
FGF21 iAUC	1.403	1.065, 1.849	0.016

Model 1: without adjustment. Model 2: Based on Model 1, further adjusted for sex, age, SBP and WC. Model 3: Based on Model 2, further adjusted for TG, HDL-C, LDL-C, HOMA-IR, IL-6.

## 4 Discussion

At present, clinical research on MAFLD focuses on exploring fasting levels. Numerous studies have found a close correlation between MAFLD and fasting serum FGF21 (11–13). However, the relationship among postprandial FGF21, MAFLD, and postprandial metabolic status remains unclear. Through an oral high-fat meal clinical trial, we found that serum FGF21 levels were significantly higher in the MAFLD population. FGF21 showed a biphasic trend of first decreasing and then increasing during the OFTT, and the amount of FGF21 decreased compared to that in the control group. MAFLD is characterized by dyslipidemia and an excessive inflammatory response after high-fat meals, which are linearly correlated with changes in postprandial FGF21. Further analysis revealed that postprandial FGF21 changes indicated an independently influenced MAFLD. Therefore, the abnormal regulation of postprandial FGF21 may be an important mechanism in the occurrence and development of MAFLD.

The present study showed that fasting FGF21 was significantly elevated in patients with MAFLD, but postprandial FGF21 changes were reduced compared to those in non-MAFLD patients, suggesting the presence of FGF21 resistance or impaired FGF21 regulation in the MAFLD population (14, 15). In this study, fasting FGF21 was significantly positively correlated with BMI and WC. Previously Alves et al. found a significant positive correlation between serum FGF21 response and BMI after sucrose intake (16). Nakanishi et al. aimed to investigate specific conditions that might influence the functions of FGF21, they found FGF21 levels correlated with age, BMI, WC and so on (17). They are consistent with our conclusion. Nutrient intake regulates serum FGF21 levels (18). In this study, the MAFLD population was administered as an oral high-fat test meal, and it was found that FGF21 levels were higher than those in the non-MAFLD population at all-time points, and that FGF21 showed a biphasic change with a decrease followed by an increase after meals. Previous studies on postprandial FGF21 levels have reported different results. Vamvini et al. reported an oral lipid load in healthy adults and found that postprandial FGF21 levels did not change significantly (19). Another study conducted an oral high-fat meal (450 kcal) trial in different age groups and found a gradual decrease in FGF21 levels (8). We believe that the differences in research results were related to the composition and calories of high-fat meals, which in this study consist of a mixed meal containing a variety of nutrients, such as carbohydrates and proteins that had a high calorie content (1500 kcal). This study found that patients with MAFLD had post prandial dyslipidemia by measuring blood lipid levels after a high-fat test meal, which is consistent with previous studies (20, 21). Postprandial serum TC, TG, FFA, and LDL-C levels were significantly elevated in the MAFLD population, and postprandial changes in TG levels were significantly higher than those in the non-MAFLD population. An excessive inflammatory response after meals in patients with MAFLD was observed by examining the levels of inflammatory factors. The study showed that IL-6, TNF-α, and hs-CRP levels were significantly higher in the MAFLD population after a test meal, and serum IL-6 was the only inflammatory indicator that underwent sustained postprandial changes. This is consistent with the results of previous studies on changes in postprandial inflammatory factors in obese individuals (22, 23).

Further analysis revealed that changes in postprandial FFA levels were associated with changes in serum FGF21 levels. Previous studies have found that changes in the circadian rhythm of FGF21 in healthy individuals are related to the FFA circadian rhythm (24). In animal experiments, Chen et al. found that growth hormone-induced hepatic production of FGF21 was achieved through the release of FFA from lipolysis (25). Mai et al. cultured HepG2 cells with different FFAs (26), which found that FGF21 showed different degrees of elevation, and further knockdown of the PPARa gene showed a decreasing trend in FGF21 levels. The result suggested that FFA can stimulate FGF21 secretion by activating the transcription factor PPARα, which provided a possible theoretical basis for the results of our study. In addition, in this study, there was a linear correlation between postprandial TG and FGF21 changes, which was confirmed in a previous study by Matikainen et al. (27) who found that postprandial triglyceride-rich lipoprotein was associated with postprandial FGF21. This may be related to the internal transformation of FFA and TG. Chronic low-grade inflammation in the body can be induced by a high-fat diet related to the pathogenesis of metabolic diseases (28–31). Abnormal postprandial metabolism is associated with chronic systemic low-grade inflammatory diseases, including atherosclerosis, diabetes, obesity, and NAFLD (32, 33). This study found that changes in IL-6 levels after meals were associated with changes in serum FGF21 levels in an MAFLD population after adjusting for age, WC and so on. Previous studies have found that fasting FGF21 is strongly associated with IL-6 in NAFLD after adjusting for multiple risk factors. Even after further adjustment for fasting CRP, fasting FGF21 remained linearly associated with IL-6 (34). Yu et al. applied a mouse model of arthritis to validate that FGF21 can suppress inflammation by activating Nrf2 and NF-κB signaling pathway (35). Yang et al. established CCl4 -induced acute liver injury models in C57BL/6 mice and the L02 cell line. The results revealed that FGF21 reduced IL-6 levels and improved inflammatory damage by enhancing SIRT1-mediated autophagy (36). Further research found that postprandial FGF21 regulation was impaired in MAFLD populations. After adjusting for age, sex, WC and other possible influencing factors, a change in postprandial FGF21 was an independent influencing factor of MAFLD. Therefore, postprandial FGF21 may be an important target for intervention against the occurrence and development of MAFLD.

To the best of our knowledge, this is the first large-sample study to determine the relationship between FGF21 and lipids, inflammation and MAFLD in the postprandial period. However, it is important to acknowledge certain limitations to this study. First, the focus of this study was to clarify the relationship between FGF21 and lipid metabolism. Therefore, the effects of blood glucose and insulin on postprandial FGF21 were not analyzed during the trial. Second, the high-fat test meal used in this study had a higher calorie intake. Hence, our team will conduct further clinical trials to investigate the optimization of the OFTT to investigate the response of FGF21 to different types of caloric diets as well as the serological factors that may influence it. We received approval from the China Natural Science Foundation and registered the study in the China Clinical Trial Registry (registration number: ChiCTR2100048497).

## 5 Conclusions

This study identified dyslipidemia and excessive inflammation during the post-meal phase in individuals with MAFLD; both of these conditions correlated with postprandial FGF21 levels. Furthermore, postprandial change of FGF21 was an independent influencing factor of MAFLD. This suggests that improving the postprandial FGF21 response may be beneficial for MAFLD, providing a possible mechanism and target for its prevention and treatment.

## Data availability statement

The raw data supporting the conclusions of this article will be made available by the authors, without undue reservation.

## Ethics statement

The studies involving humans were approved by The Ethics Committee of Hebei General Hospital. The studies were conducted in accordance with the local legislation and institutional requirements. The participants provided their written informed consent to participate in this study. Written informed consent was obtained from the individual(s) for the publication of any potentially identifiable images or data included in this article.

## Author contributions

XL: Data curation, Methodology, Writing - original draft, Investigation, Validation, Writing – review & editing. KZ: Data curation, Formal analysis, Writing – review & editing. LL: Formal analysis, Investigation, Methodology, Writing – review & editing. TZ: Software, Supervision, Writing – review & editing. WG: Project administration, Validation, Visualization, Writing – review & editing. XH: Conceptualization, Project administration, Software, Writing – review & editing. JG: Formal analysis, Investigation, Supervision, Writing – review & editing. GS: Conceptualization, Data curation, Funding acquisition, Methodology, Project administration, Resources, Writing – review & editing.
