# Supplementary figures and images for: Relationship of postprandial fibroblast growth factor 21 with lipids, inflammation and metabolic dysfunction-associated fatty liver disease during oral fat tolerance test
Source: Front Endocrinol (Lausanne). 2024 May 17;15:1343853. doi: 10.3389/fendo.2024.1343853 (PMC11140040; doi:10.3389/fendo.2024.1343853)

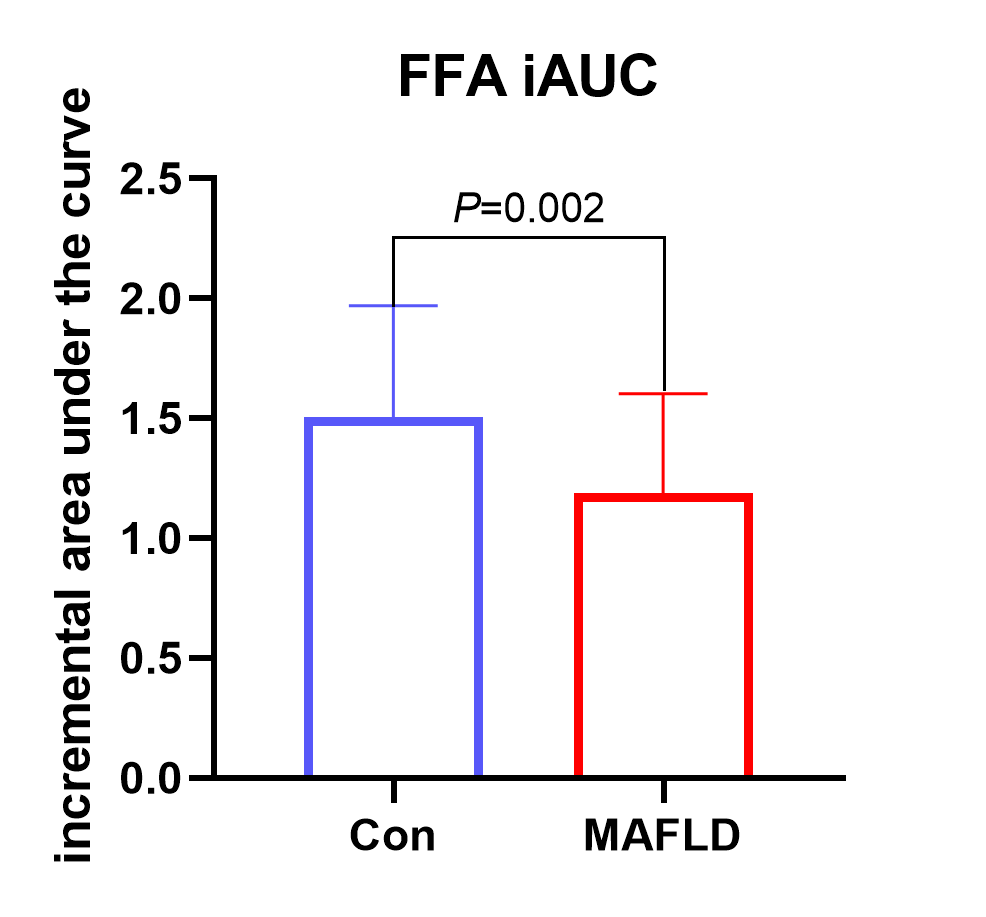

Supplement: Supplementary file 1 [file Image_1.tif]

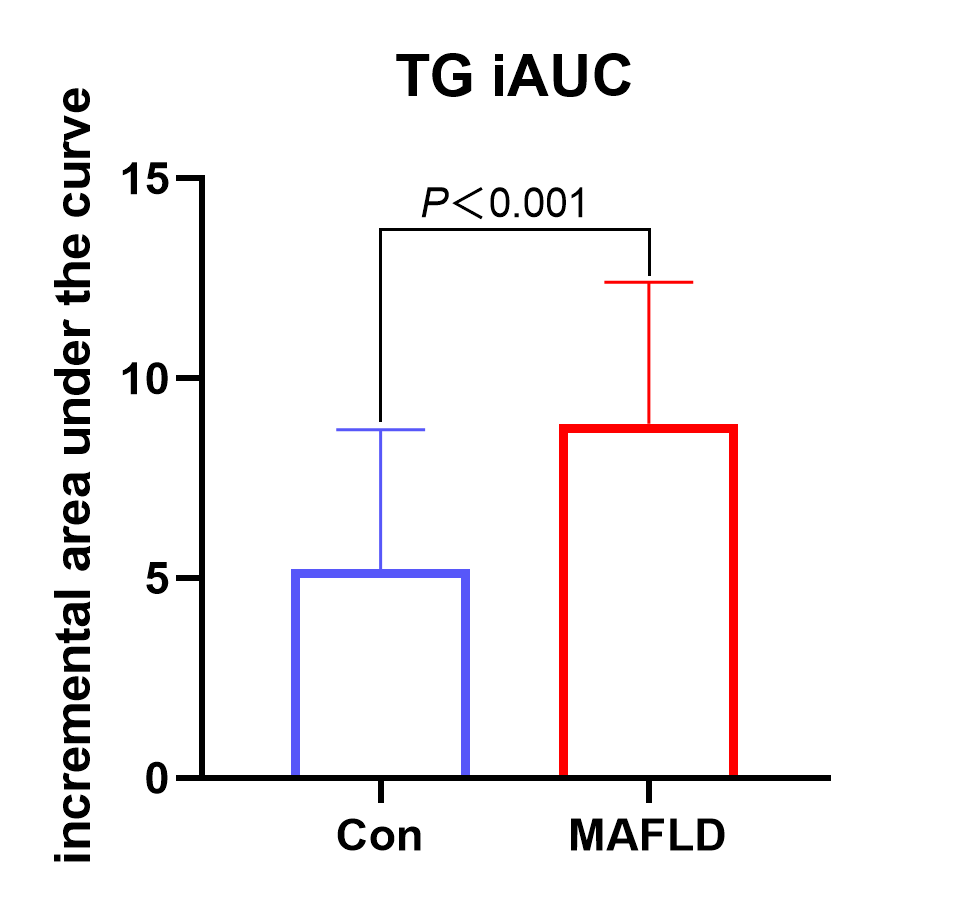

Supplement: Supplementary file 2 [file Image_2.tif]
